# Mind-wandering in China and the UK: evidence of cross-cultural consistency

**DOI:** 10.3389/fpsyg.2026.1715597

**Published:** 2026-03-03

**Authors:** Qiuyu Du, Andrew Tolmie, Rebecca Gordon

**Affiliations:** Department of Psychology and Human Development, Institute of Education, University College London, London, United Kingdom

**Keywords:** mind wandering, meta-awareness, self-generated thought, cross-cultural variation, causes of mind wandering

## Abstract

**Introduction:**

Mind wandering has been studied using various operational definitions, resulting in conceptual inconsistencies. Understanding whether it reflects universal cognitive patterns or culturally shaped experiences is critical for theories of attention and self-regulation.

**Methods:**

A total of 17 British and 32 Chinese participants completed a monotonous writing task designed to provoke mind wandering, followed by a semi-structured interview probing the content and contexts of their thoughts.

**Results:**

Results revealed that mind wandering characteristically involves self-generated thoughts, with participants frequently aware of these and allowing them to continue. No significant cultural differences were found in the frequency or causes of mind wandering. However, British participants more frequently described its effects as non-negative, whereas Chinese participants emphasised negative outcomes.

**Discussion:**

This variation possibly reflects cultural values that prioritise effortful attention as a moral duty. This highlights the interplay between universal cognitive processes and cultural influences.

## Introduction

Interest in mind wandering has grown in recent years (see Kawashima et al., 2023 for a meta-analysis), with research in this area characterised by a range of operational definitions, particularly task-unrelated thought (TUT), stimulus-independent thought (SIT), and spontaneous thought, and these are sometimes used interchangeably. The current study attempted to identify the common characteristics of everyday experiences of mind wandering in order to understand participants’ perspectives on their mind wandering experiences and identify common characteristics that could inform a more consistent definition of its key elements. The study also aimed to understand whether these elements are innate or influenced by cultural factors.

### Definitions of mind wandering

Mind wandering refers to a shift in the mental train of thought away from the demands of current and purposeful activity. Thoughts that emerge during mind wandering experiences have frequently been characterised as *task-unrelated* or *stimulus-independent*. Both terms emphasise that the experience is separate from ongoing mental processing related to a current task, but in different ways. TUT directs attention away from the current task or situation (e.g., thinking about an upcoming holiday while listening to a lecture), while SIT refers to thought that is decoupled from current sensory information (i.e., independent of any particular stimuli related to the current task or situation) (Smallwood et al., 2004; Teasdale et al., 1993). To further explain, SIT can be unrelated to the current task (e.g., chopping something while cooking), but related to the task as a whole (e.g., planning the next steps of a recipe while chopping). TUT, by contrast, is thought which is not task-related, whether directed inward (e.g., thinking about an upcoming holiday) or outward (e.g., thinking about a bird outside).

*Spontaneous thought* focuses on how mind wandering arises rather than its relationship to a current, purposeful activity. It can be defined as unintentional mental content that arises without immediate awareness, emerging when cognitive constraints are minimal (Christoff, 2012; Christoff et al., 2016). Spontaneous thought emerges when both deliberate and automatic cognitive constraints are reduced (Christoff et al., 2016). It is characterised by the absence of deliberate intention, distinguishing it from deliberate forms of TUT or SIT (e.g., choosing to tune out during a boring lecture). However, when TUT or SIT occur automatically (e.g., unintentionally zoning out), they may qualify as spontaneous thought. Table 1 captures the similarities and difference between the three constructs resulting in seven types of mind-wandering.

**Table 1:** A conceptual framework distinguishing task-unrelated thought (TUT), stimulus-independent thought (SIT), and spontaneous thought.

Task relation/intentionality	Stimuli type	
	Internal stimulus	External stimulus
Task related		
Deliberate	SIT	
Unintended	SIT/spontaneous thought	Spontaneous thought
Task unrelated		
Deliberate	SIT/TUT	TUT
Unintended	SIT/TUT/spontaneous thought	TUT/spontaneous thought

### Evidence from neuroscience

Imaging studies support the separability of the three constructs. For TUT, functional magnetic resonance imaging (fMRI) shows three regions of the brain. These are the medial prefrontal cortex, the posterior cingulate/praecuneus region and the temporoparietal junction (widely referred to as the default network; Christoff et al., 2009). These regions are more active when participants experience TUT compared to when they are on task (Mason et al., 2007). For SIT, the medial prefrontal cortex region is more specifically implicated (Mason et al., 2007). The medial prefrontal cortex has also been linked to spontaneous thought, but Spiers and Maguire (2006) observed the further contribution of brain regions outside the default network, especially the temporopolar cortex. Given that only partially overlapping activity is observed in TUT, SIT and spontaneous thought (i.e., the medial prefrontal cortex), it is possible they have some degree of functional as well as conceptual separability.

Given these definitions and related neuroimaging evidence, coupled with the various terminology in the broader literature, there is a need for a more detailed definition or taxonomy of mind wandering that encompasses the dimensions covered by TUT, SIT and spontaneous thought. In pursuit of this, it is important to understand the types of mind wandering that people actually experience on an everyday basis, rather than simply those that are conceptually possible. In doing so, future work can investigate key dimensions of mind wandering phenomena in various contexts. The current study research therefore used task-stimulated semi-structured interviews to capture participants’ subjective experience of mind wandering, including its typical content and when it occurs.

### Cultural differences in mind wandering

Given the focus on actual experience of mind wandering, it is also important to consider the extent to which this exhibits cultural variation. Mind wandering is a ubiquitous phenomenon which has been studied in many countries, including the United Kingdom (Seli et al., 2018), China (Song and Wang, 2012), Japan (Yamaoka and Yukawa, 2020), and Brazil (Gonçalves et al., 2017), as well as extensively within America (Killingsworth and Gilbert, 2010; Schooler et al., 2011; Wegner, 2018). Cultural differences in its occurrence and content have been observed. Sude (2015) tested whether there is a difference in the rates of mind wandering between the European-heritage and the Asian-heritage samples using a Sustained Attention to Response Task with periodic thought probes to capture current mental activity. The task is characterised by its monotonous, high-frequency Go/No-Go demands, which impose a heavy load on sustained executive control. The results showed that the Asian-heritage sample reported fewer incidents of mind-wandering, though the types of thoughts experienced were similar. In contrast, Gonçalves et al. (2017), using the Attention Network Task (ANT), failed to find differences between Portuguese (southern European heritage) and Brazilian (southern American heritage) participants in terms of either rate or general content of mind wandering. The ANT, which intermixes alerting, orienting, and executive conflict trials, presents a more varied and complex set of attentional challenges. However, Portuguese participants reported more task-related thoughts (e.g., mentally rehearsing their responses or counting how many trails remained) that interfered with task performance. This divergence in findings likely underscores the fundamental influence of task paradigm on the measurement of mind-wandering. Crucially, in both studies, the interpretation of self-reported mind-wandering is confounded by task performance. For instance, Portuguese participants’ increased reporting of performance-related thoughts in the ANT may reflect a strategic adaptation to the task’s specific challenges, rather than a pure difference in spontaneous mind-wandering propensity.

Such cultural differences may be rooted to some extent in the broader cultural dimensions of individualism and collectivism (Hofstede and Hofstede, 2000). An individualistic cultural orientation leads to a focus on individuals’ personal goals, while a collectivist cultural orientation emphasises relationships with other members of the society. Collectivism is dominant in East Asia, where group concerns (e.g., group harmony and cohesion) are placed above individual concerns (e.g., self-improvement). Individualism, which is more dominant in Western Europe and North America emphasises the separation of the self from others and places group concerns below individual concerns. These cultural differences could explain why Asian-heritage participants in Sude’s (2015) study reported less frequent mind wandering compared to the European-heritage sample. That is, the greater focus on group concerns may result in greater concentration on externally set tasks, and hence fewer incidents of mind wandering, but with similar thought content to the European sample when it did occur. Individualistic orientations (as might be seen in European participants) could show more context-dependent engagement, increasing focus when tasks align with personal goals or decreasing focus when task feel externally imposed. The collectivist framework possibly treated the task as a social commitment, whereas individualists may have engaged more selectively. Conversely, the lack of difference reported by Gonçalves et al. (2017) may be attributable to European and American nations having similar individualistic cultural values.

The greater concern of Portuguese participants (compared to their Brazilian counterparts) with thoughts that interfered with the current task may suggest another potential dimension of cultural variation, which might be based on broader cognitive processing styles (Nisbett et al., 2001). Eastern cultures, such as China, are often associated with a holistic style, while Western cultures emphasise analytical thinking. Research suggests Brazilians exhibit relatively holistic thinking styles, prioritising context, relationships, and flexibility, even compared to other Western groups (De Oliveira and Nisbett, 2017). Despite sharing a colonial history, Portuguese groups might retain a more analytic cognitive style, which is characterised by a focus on the specific details and components of a situation rather than broader relationships between elements and context. This could explain why they experienced more interfering thought as analytic thinkers tend to prioritise focal tasks over contextual distraction.

These cognitive differences appear not just in thought patterns but in perceptual processes. For instance, eye-tracking studies (e.g., Chua et al., 2005; Cenek et al., 2020; Goh et al., 2013) have shown that individuals from East Asian cultural backgrounds tend to allocate visual attention more broadly, while those from Western backgrounds focus more narrowly on salient objects. This perceptual tendency may establish a habitual attentional framework that could influence the dynamics of internal thought. However, it is crucial to distinguish between this general attentional disposition and the specific, endogenous event of mind-wandering.

Given these conflicting findings and the highly speculative nature of the underlying mechanisms, a more focused empirical investigation is needed. The present study was designed as an exploratory investigation to provide clearer evidence on whether group-level differences in mind-wandering experience exist between cultures known to vary on the dimensions of individualism–collectivism and analytic-holistic thinking. To this end, we examined individual experiences of mind-wandering in Chinese and UK nationals. While we acknowledge the likelihood for significant within-country variation, this comparison was carefully selected based on the established theoretical contrasts outlined above. Our aim was to understand, in greater detail, any cultural differences in terms of when mind wandering occurs, awareness of its occurrences, and individual attitudes towards its value, providing a foundation for future research with more precise cultural measurement.

### The current study

The current study used samples from both Britain and China as typical examples of individualist/ analytical and collectivist/relational cultures, respectively. The study investigated the characteristics of participants’ mind wandering experiences to provide the basis for an alternative taxonomy. The taxonomy organised mind wandering experiences into four key dimensions: reasons, content, meta-awareness, and effects, which extends the concept of mind wandering beyond simple task-related classification to capture the full complexity of experiences. The use of an unappealing and easy task aimed to increase rates of mind wandering (Seli et al., 2015; Unsworth and McMillan, 2013). Participants were asked to record their thoughts when they made errors during this task (indicating straying of attention), and thought probes further sampled the frequency of mind wandering. Participants were then interviewed about their real-world experiences of mind wandering. General patterns and group comparison were made with regard to the incidence, triggers and content of mind wandering.

## Methods

The present study selected samples from the United Kingdom and China as comparative cultural groups. This selection was guided by the theoretical frameworks discussed in the introduction, where these nations are frequently cited in the literature as exemplars of cultures that differ on the broad dimensions of individualism–collectivism and analytic-holistic cognition (e.g., Nisbett et al., 2001; Hofstede and Hofstede, 2000). Our approach was explicitly exploratory: we hypothesised that if gross, group-level differences in the phenomenology of mind-wandering were linked to these overarching cultural contexts, they would be detectable even in the absence of fine-grained individual measures of cultural orientation. We acknowledge that there is significant within-country variation in both the UK and China, and that without direct measures of individual cultural values or cognitive styles, we cannot attribute any observed differences definitively to specific cultural dimensions. Consequently, findings will be interpreted cautiously as suggestive patterns that justify and inform future research with more controlled designs and validated cultural instrumentation.

### Participants

From 86 potential participants 49 met the inclusion criteria (i.e., born and brought up in the China or UK as native Chinese or English speakers respectively). Participants were recruited through university participant pools and social media advertisements targeting university students in the UK and China. The sample comprised 17 participants from Britain and 32 from China. See Table 2 for sample characteristics. There were no significant differences between the two groups in terms of age (*t*(47) = 1.50, *p* = 0.14), or sex (χ^2^(1) = 1.87, *p* = 0.17). The disparity in group size resulted from practical recruitment challenges, notably those associated with the COVID-19 pandemic context during the data collection period. Despite this imbalance, thematic saturation was achieved for the core qualitative analysis within each cultural group. All participants provided informed consent. The sample size was deemed sufficient to meet the principle of saturation (cessation of data collection when no new patterns emerge), commonly employed in qualitative research (Ahmed, 2025). Data collection was conducted online via Zoom and WeChat. To ensure careful monitoring and recording of participants’ behaviours and responses, during a writing task participants were asked to position their cameras to focus on their answer sheets and reposition to focus on themselves during an interview. Thirty-three participants provided specific consent for this experimental procedure to be recorded (*n* = 16 Chinese). Of these, eight Chinese participants also agreed to the use of a voice recorder. For a further eight participants who did not agree to electronic records being kept, key points in the interviews were recorded by the researcher using pencil and paper. The study was approved by the authors’ institutional Research Ethics Committee.

**Table 2:** Sex and age mean of the British and Chinese groups.

Group, sex	*N*	Mean age (SD)
British		
Female	15	33.93 (12.13)
Male	2	22.00 (4.24)
Chinese		
Female	20	27.6 (9.07)
Male	12	28.00 (11.32)

### Materials and procedure

A simple task was employed to provide a recent, structured, and shared experiential context to ground the subsequent in-depth interview. The primary goal was not to sample mind-wandering with high ecological validity for quantitative analysis, but to create a concrete referent from which participants could draw detailed, specific reflections during the qualitative phase. The number transcription task was selected for its simplicity and low cognitive demand, which was intended to increase the likelihood of attentional lapses without requiring complex instructions or creating performance anxiety that could interfere with later discussion. Participants were instructed to write the numbers from 1 to 400 on a sheet of paper. Due to its simplicity, it was assumed that any errors that appeared in the process of writing were the result of a lapse in concentration (rather than due to task complexity), possibly due to incidents of mind wandering. Participants were asked to highlight any self-identified errors on the paper and also make note of their concurrent thoughts in a mistake log. This was then referenced later for discussion during the interview phase. To further probe the incidence of mind wandering, participants were randomly interrupted by the researcher on two to eight occasions to answer the question, “what were you just thinking about?.” This dual-method approach—combining self-caught errors and researcher-initiated probes—was designed to capture a broader spectrum of mind-wandering episodes for later discussion, including those that occur without the participant’s meta-awareness and thus would be absent from purely retrospective or self-caught reports. The number of interruptions varied according to participants’ performances. Participants were interrupted more often if they made more than 4 errors, or if it could be seen on camera that the writing process contained significant pauses (i.e., more than 5 s) or hesitations. The task lasted approximately 18 min for each participant, dependent on number of interruptions.

Immediately after completing the writing task, participants participated in a semi-structured interview, with the camera repositioned to focus on the participants. The interview consisted of two parts. First, participants were asked to recall and describe what they were thinking about when they made errors in the writing task, using either the notes they had recorded in their mistake logs or the marks they made on their worksheets. Next, participants were presented with a series of open-ended questions about similar experiences in their daily lives. In order to reduce bias, the term mind wandering was not used in the questions, instructions or information sheet. The interview questions were derived from gaps identified in the literature concerning the subjective phenomenology of mind-wandering (e.g., awareness). These questions were then refined through pilot interviews with a small number of participants (*n* = 4) not included in the main study. The pilot phase tested question clarity, flow, and their effectiveness in eliciting rich, relevant descriptions of experience. The final set of four open-ended questions was selected to comprehensively cover the core dimensions of interest while minimising researcher bias and leading language.

Prior to the interview questions, participants were given the following preamble to orient them to discussing mind wandering experiences: “Earlier you did a task where you might have noticed your thoughts shifting away from the numbers. We are interested in these kind of experiences—when your attention moves to other things. Think about times this has happened, and then answer these questions”. The questions were as follows: (1) In what circumstances do you tend to experience such mental activity? (2) What makes it more likely to happen? (3) How do you think this mental activity will affect the task you were doing? (4) Did you know that you were experiencing this mental activity? and if so, did you let this mental activity continue deliberately? Each interview lasted approximately 30 min.

### Data analysis

Grounded theory (Glaser and Strauss, 2017) was used to analyse the interview responses. This method was chosen as it is appropriate when developing a theory directly from qualitative data, as opposed to *a priori* development of the theory. The grounded theory analysis progressed through three key stages while maintaining constant comparison throughout the process. In the initial open coding phase, raw participant responses were broken down and assigned descriptive labels to each meaningful segment. For instance, when participants expressed physical discomfort like “I feel so tired, I really do not want to write!” or “My thighs are so itchy,” the discrete codes “Tired” and “Itchy” were created, respectively.

The axial coding stage then organised these codes by grouping them into broader conceptual categories. The codes “Tired” and “Itchy” clustered together under the axial category “Physical feelings.” These physical states frequently coincided with reports of task disengagement or attention shifts. This systematic comparison allowed us to verify that physical discomfort consistently emerged as a meaningful factor influencing participants’ focus.

The final selective coding stage integrated these axial categories into the core theoretical framework. The recurrence of physical feelings as attention disruptors, particularly during prolonged tasks, became one of the key insights in the understanding of mind-wandering triggers. To ensure the trustworthiness of the qualitative analysis, several established strategies for enhancing credibility in single-researcher qualitative studies were employed. First, the process was highly iterative, that is, all transcripts were coded multiple times over an extended period (approximately 4 months), with each round of coding checking for consistency with previous rounds and refining category definitions. Second, negative case analysis was conducted. Instances that did not fit the developing taxonomy were specifically examined to refine category boundaries and ensure the framework was comprehensive rather than selectively representing the data. Finally, to enhance the credibility of the emerging categories, a process of peer debriefing was conducted: preliminary codes and thematic structures were discussed regularly with two research colleagues not directly involved in the study, who challenged assumptions and provided alternative interpretations. The coding process are summarised with examples in Table 3.

**Table 3:** Coding process from raw data to core categories.

Stage	Process	Data exemplar and progression
Open coding	Breaking down raw data into discrete codes	Exemplar Set A (Physical Triggers): “I feel so tired, I really do not want to write! When will it end?” → Code: Tired
		“My thighs are so itchy. I want to scratch them so badly.” → Code: Itchy Exemplar Set B (Task Attitudes): “This is incredibly boring.” → Code: Boring “It’s so easy, my mind checks out.” → Code: Too easy
Axial coding	Grouping open coded into categories that explain why a phenomenon occurs	From Exemplar Set A: Codes: Tired, Itchy Hungry → Axial Category: Physical Feelings Concept: Bodily states that disrupt focus From Exemplar Set B: Codes: Boring, Too Easy → Axial Category: Attitude to Task Concept: Perceptions of the task that promote disengagement Other Formed Categories: Nervous, Stressed, Worried → Emotional State Noise, Conversation, Machinery → External Sounds
Selective coding	Integrating categories into core theory	Axial categories: “Physical feelings,” “Attitude to task,” “Emotional state,” “External sounds” → Core Category: Reasons for mind wandering

Once this analysis had generated a mapping of participants’ mind wandering experience, it became necessary to determine whether the observed patterns differed between the two cultural groups. To test it, the data were examined using quantitative methods. For any differences between the two cultural groups. Mann–Whitney tests and correlational analysis were used to examine groups differences and relationships between observed indices (e.g., errors count and codes frequencies).

Interviews were conducted in the participants’ native languages: English for British participants and Mandarin Chinese for Chinese participants. All Mandarin interviews were transcribed verbatim and then translated into English by a bilingual researcher fluent in both languages and familiar with psychological terminology. Coding was conducted primarily by one bilingual researcher who had lived experience in both cultural contexts. Discrepancies were resolved through discussion, and ambiguous or culturally nuanced expressions were reviewed with an additional bilingual consultant.

## Results

Performance on the number-writing task provided the behavioural context for the subsequent qualitative analysis. Participants made an overall mean of 3.94 errors per person (SD = 1.82, range = 1–8; see Table 4). The British group averaged numerically but not significantly higher at 4.12 errors (SD = 1.95, range = 1–8) compared to the Chinese group’s mean of 3.82 errors (SD = 1.75, range = 1–7), *z* = −1.39, *N_C_* = 32, *N_B_* = 17, *p* = 0.17. The researcher also initiated thought probes to capture moments that might not result in a self-caught error. British participants (*M* = 3.29, SD = 0.69) received significantly more probes than Chinese participants (*M* = 2.34, SD = 0.90), *t*(47) = 4.04, *p* < 0.001, consistent with the adaptive protocol that administered more probes following errors or pauses. These procedures yielded two primary sources of in-task thought reports for the qualitative analysis. Participants’ descriptions of their thoughts at the moment they logged an error (mind wandering with awareness); and thoughts reported in response to researcher interruptions (mind wandering without awareness). In terms of reported mind wandering frequency, there were no significant differences between the Chinese (*M* = 1.75, SD = 1.46) and British (*M* = 1.65, SD = 1.17) groups, *t*(48) = −0.25, *p* = 0.80. The frequency of mind wandering with awareness thoughts reported at the moment a self-caught error was logged. This did not differ between British (*M* = 3.18, SD = 1.59) and Chinese (*M* = 3.47, SD = 1.48) groups, *t*(47) = −0.64, *p* = 0.53. The frequency of mind-wandering without awareness episodes reported in response to researcher-initiated probes. This also showed no group difference (British: *M* = 0.94, SD = 1.56; Chinese: *M* = 0.66, SD = 0.75; *t*(19.96) = 0.71, *p* = 0.49).

**Table 4:** Number of times participants made mistakes in the task.

Group	Mean errors	SD	Range	*N*
General	3.94	1.82	1–8	49
British	4.12	1.95	1–8	17
Chinese	3.82	1.75	1–7	32

Reasons for mind wandering included four factors: external sound, attitude to task, physical feelings, and emotional states. The content of mind wandering was broken down into two categories based on: whether thoughts were related or unrelated to the task. For meta-awareness, mind wandering was divided into two categories: mind wandering with and without awareness. In terms of effect, participants reported neutral, positive, and negative outcome of mind wandering. Descriptions of the open and axial coding categories under each core category can be found in Table 5, specifically focusing on participants’ subjective experiences and evaluations of mind-wandering.

**Table 5:** The results of coding mind wandering experiences (the number in parentheses indicates the number of times the code occurred).

Core categories	Axial coding	Open coding
Reason	External sounds (44)	Noise from streets, conversations in another room, hums of machinery
	Attitude to task (48)	unappealing task (boring, difficulty, easy, too long, repetitive)
	Physical feelings (21)	Itchy (5), tired (16)
	Emotion state (39)	Nervous (9), careful (14), uncomfortable (2), stressful (11), Worried (2), Perfect (1)
Content	Unrelated to task (141)	Leisure activity (43), work or study (38), personal relationship (31), self issue (5), others (24)
	Related to task (128)	Performance (28), process (36), subsequent task (13), previous task (16), others (35)
Meta-awareness	With awareness (150)	With awareness and let continue (87), with awareness and stop (63)
	Without awareness (48)	Not actively notice this mental shift as it happens
Effect	Negative effect (28)	Impair performance (3), reduce efficiency (18), unconcentrated (3), delay learning (6)
	Positive effect (8)	Refresh mind (4), feel relieved (4)
	Neutral (13)	Neither embrace nor reject mind wandering experiences

### When and why do people’s minds wander?

Participants demonstrated variable error rates during the task. When asked about these specific error moments in the interviews, participants most frequently attributed their lapses to internal, self-generated states. Qualitative analysis revealed that 71% of reported triggers were internal, supporting a definition of mind wandering centred on internal thought generation.

Analysis of interview responses revealed five primary reasons for mind wandering. These were:

- Task attitudes (31.6%): Most common was negative task perception (e.g., “This is so boring”).
- External distractions (28.9%): Environmental interruptions like traffic noise.
- Emotional states (25.7%): Stress or nervousness unrelated to the task.
- Physical feelings (13.8%): Bodily discomfort (e.g., fatigue, itchiness).

### What happens during mind wandering?

The analysis of thought content during attentional lapses revealed a near-even distribution between task-unrelated (52.4%) and task-related thoughts (47.6%). Within task-unrelated content, three primary subcategories emerged: leisure activities accounted for 16% of instances (e.g., “planning weekend activities”), work/study-related thoughts comprised 14.1% (e.g., “remembering an unfinished project”), and personal relationships represented 10.8% (e.g., “thinking about family members”). The proportion of thoughts related to the current task (47.6%), included aspects such as thoughts about the previous task, the subsequent task, performance, process, and other task-related issues. Task process was the most common of these (13.4%).

### Are people aware of their states of mind wandering?

With regard to meta-awareness, participants most often (75.76%) reported that they were aware of their mind wandering during a task, and commonly let their minds continue to wander (43.9%). Comments which supported this include: “When I am attending a lecture and the topic is difficult and boring, I would like to think of something to pass the time,” and “I deliberately recall the game that I played particularly well.”

### What are peoples’ opinions of the consequences of mind wandering?

Despite the tendency to let mind wandering experiences continue, the responses on effect were focused on its negative effects (57.1%), such as a reduction in efficiency, interference with progress, and hindrance to the learning process. Some of the sample (26.5%) were unsure of the impact of mind wandering, expressing a neutral attitude towards it. Only a small number (16.3%) mentioned positive effects, such as a means of mental relaxation, and facilitating enhanced concentration on subsequent tasks.

### Cultural differences in mind wandering

The following quantitative comparisons are exploratory in nature, given the sample size limitations noted in the Methods. They are presented to provide a descriptive context for qualitative themes. In terms of reasons for mind wandering (see Table 6, percentages represent proportion of total cause reported within each cultural group), Mann–Whitney tests showed there were no significant differences between the British and Chinese groups in terms of external noises, *z* = −0.94, *N_C_* = 32, *N_B_* = 17, *p* = 0.35, attitude to task, *z* = −1.50, *N_C_* = 32, *N_B_* = 17, *p* = 0.14, physical feelings, *z* = −1.89, *N_C_* = 32, *N_B_* = 17, *p* = 0.06, or emotional state, *z* = −1.63, *N_C_* = 32, *N_B_* = 17, *p* = 0.10.

**Table 6:** Causes of mind wandering in British and Chinese participants.

Categories of mind wandering causes	External noises	Attitude to tasks	Physical feelings	Emotional state
British	31.82%	28.79%	22.73%	16.67%
Chinese	40.58%	28.99%	15.94%	14.49%

When examining the content of task-unrelated thoughts (see Table 7), there were no significant differences between the British and Chinese groups in terms of a focus on work or study (*z* = −1.48, *N_C_* = 32, *N_B_* = 17, *p* = 0.14); on family or friends (*z* = −0.72, *N_C_* = 32, *N_B_* = 17, *p* = 0.47); or for those categorised as “other” (*z* = −0.15, *N_C_* = 32, *N_B_* = 17, *p* = 0.88). However, there was a significant difference in thoughts about leisure activity (*z* = 2.58, *N_C_* = 32, *N_B_* = 17, *p* = 0.01), with the Chinese group (*M* = 1.03, SD = 0.99) reporting this more frequently compared to the British group (*M* = 0.35, SD = 0.70). The Chinese and British groups did not differ significantly in occurrence of task-related mind wandering (*t*(48) = 0.58, *p* = 0.56).

**Table 7:** Types of thought content in British and Chinese participants during mind wandering.

Types of thought content	Task related Information	Task unrelated information			
		Leisure activity	Work or study	Family or friends	Others (e.g., self, daily issues etc.)
British	51.04%	6.52%	20.83%	13.54%	8.07%
Chinese	51.23%	20.37%	8.64%	12.35%	7.41%

In terms of the effects of mind wandering (see Table 8), 75% of Chinese participants held the view that mind wandering had negative consequences, with only a small number reporting positive or neutral effects. In contrast, British participants had a more diverse range of opinions about the consequences of mind wandering. A chi-squared test found a significant association between nationality and participants’ beliefs about the effects of mind wandering (χ^2^(2) = 15.53, *p* < 0.01). While both groups recognised the negative consequences of mind wandering, British participants also noted positive and neutral outcomes.

**Table 8:** Participants’ perceptions of mind wandering effects in British and Chinese participants.

Participants’ perceptions	Negative effects	Positive effects	Neutral effects
British	17.65%	23.53%	58.82%
Chinese	75%	3.13%	21.87%

### Relationships between themes and task performance

To directly link qualitative themes with quantitative measures, we conducted Spearman correlations and non-parametric comparisons. Emotional states as a trigger for mind wandering was positively correlated with error counts (*r* = 0.35, *p* = 0.02) but not with mind wandering frequency (*r* = 0.06, *p* = 0.70). Mind wandering with and without awareness was unrelated to errors (*Z* = 1.22, *N*_with_ = 45, *N*_without_ = 4, *p* = 0.22), frequency (*Z* = 0, *N*_with_ = 45, *N*_without_ = 4, *p* = 1.00), and evaluation (Fisher’s Exact Test = 1.58, *p* = 0.54). To examine whether the content of mind wandering was related to task performance, we calculated for each participants the proportion of task-related thoughts and task-unrelated thoughts during mind wandering. Task-related thought proportion correlated positively with errors (*r* = 0.31, *p* = 0.03) and marginally negatively with frequency (*r* = −0.25, *p* = 0.08). Task-unrelated thought proportion correlated negatively with errors (*r* = −0.31, *p* = 0.03) and positively with frequency (*r* = 0.29, *p* = 0.05).

While emotional triggers were associated with higher error rates, they were unrelated to the frequency of mind wandering. This dissociation suggests that emotional arousal might impair performance by compromising error monitoring, slowing motor execution, or increasing impulsive responding—rather than merely causing more frequent attentional shifts away from the task. These complementary patterns suggest that mind wandering characterised by task-related content occurs less frequently but is more detrimental to performance, whereas task-unrelated mind wandering occurs more frequently but with less performance cost.

## Discussion

This study employed a mixed-methods approach with a primary focus on qualitative, exploratory analysis to develop a detailed taxonomy of mind-wandering experience. The simple behavioural task served to provoke and ground discussions for in-depth interviews, which were the core source of data. The quantitative cross-cultural comparisons are presented as secondary, descriptive observations that must be interpreted with caution due to the qualitative design and sample characteristics. By combining a behaviour task with in-depth interviews, this study provides a holistic understanding of mind-wandering. The simple quantitative task served to provoke and identify moments of attentional lapse, while the qualitative interviews richly contextualised what those lapses meant subjectively. This mixed-methods approach reveals that mind-wandering is not merely ‘task-unrelated thought’ but a complex experience with consistent dimensions across cultures. Findings indicate that mind wandering is a multifaced process, with individual differences in the perceptions of mind wandering in terms of reason, content, meta-awareness and effect. Using an interview approach and accompanying cross-cultural comparison, this study uncovered key aspects of how mind wandering is experienced and perceived.

### A taxonomy of mind-wandering

As definitions, TUT, SIT, and spontaneous thought provide valuable but partial lenses on mind wandering, each emphasising different aspects of the phenomenon. The findings confirm that participants experience all seven forms outlined in Table 1, yet no single existing construct captures the full phenomenological complexity reported. More importantly, the grounded approach reveals that these traditional frameworks systematically neglect key dimensions of subjective experience that are central to how individuals understand and evaluate their own mind wandering. The four-dimensional taxonomy supplements these existing models by adding critical experiential layers. Specifically, where TUT/SIT focus primarily on content (task-relatedness), our taxonomy adds the dimensions of Reasons (why mind wandering occurs) and Effects (how it is evaluated), capturing the motivational and evaluative context that shapes the experience. Spontaneous thought frameworks focus on constraints (deliberate vs. automatic); the taxonomy explicitly incorporates Meta-awareness, distinguishing not only how thoughts arise but also how they are monitored and regulated consciously.

The taxonomy also helps sharpen the boundary this definition draws: being distracted by outdoor noise should not be considered as an example of mind wandering. This because, in these circumstances, the mind shifts towards external distraction due to involuntary attention to novelty and changes in the environment (Escera et al., 2000). This kind of attention without conscious control is an automatic response to certain sensory inputs (Angell, 1904), such as a door slamming while someone is writing. This distinction, evidenced in the data, confirms that mind wandering is defined only when external distractions or immediate activity leads to internal processes rather than the focus remaining solely on the external event itself. Self-generated thought, defined in this way, is independent of task relatedness: it can be task related or task unrelated (Smallwood and Schooler, 2015). Task-related self-generated thoughts maintain some connection to current activities, such as when participants reflect on their experimental role while engaged in study procedures. Conversely, task-unrelated self-generated thoughts represent complete disengagement from current tasks in favour of internal mental activity, like planning future activities during experimental participation (see Table 9 for the classification of mind wandering). Mind wandering’s essential characteristic lies in its internal origin and dynamics rather than simply representing disengagement from external tasks or stimuli.

**Table 9:** The classifications of mind wandering and similar mental states.

Mode of thought	Related to task	Unrelated to task
Perceptually guided	Writing the numbers 4, 5, 6 (Task focus)	Noise of motorcycle (Distraction)
Self-generated	What is the point of this task? (Task related reasoning)	Where to go for brunch on the weekend? (Mind wandering)

### Awareness of mind wandering

Findings from this study suggest that participants were typically aware of their mind wandering state and allowed it to continue, which confounds the common conceptualisation of mind wandering as reflecting unintentional thoughts. There is a possibility that participants might have intentionally engaged in mind wandering episodes when they had little motivation to engage in the number writing task, and conscious mind wandering might not be so prevalent under other circumstances which might be assigned greater importance or be deemed more enjoyable. Nevertheless, this distinction appears to be important for understanding the subjective experience of mind wandering, as it suggests that not all mind wandering is the same.

Mind wandering without awareness may reflect a deeper level of cognitive disengagement, while mind wandering with awareness may reflect goal-directed regulation. Consistent with this, Seli et al. (2015) investigated the distinction between spontaneous mind wandering and deliberate mind wandering at a trait level. Their results revealed that spontaneous mind wandering had a negative relationship to a “Non-Reactivity to Inner Experience” dimension (as measured by the five-facet mindfulness questionnaire, whereas deliberate mind wandering showed a positive association). This finding suggests that different types of mind wandering may have different cognitive and behavioural consequences. Therefore, differentiating the level of awareness of mind wandering in future research might shed some important light on the processes involved in, and stemming from, mind wandering.

### Cultural differences in mind wandering

This study reveals a foundational cross-cultural consistency in the experience of mind wandering—with no significant differences observed in its frequency, triggers, or content. Within this overarching framework of similarity, a descriptive nuance emerged in how participants evaluated its effects: British participants more often described neutral or positive outcomes, whereas Chinese participants predominantly emphasised negative consequences. This divergence in evaluative focus likely reflects the influence of deeper cultural values, which will be discussed in the following.

Confucian values are at the heart of Chinese education, but their influence can be understood through distinct dimension. Beyond a collectivist orientation emphasising group harmony, Confucian tradition also emphasises authoritarianism in learning contexts—prioritising obedience, discipline, and respect for hierarchical relationships between student and teacher or task (Tweed and Lehman, 2002). Simultaneously, Confucian harmonious values—prioritising internal and social balance—may lead participants to view a wandering mind as disruptive to internal cognitive harmony and the harmonious engagement with a given duty. The experience of off-task thoughts conflicts with the cultural ideal of a focused and balanced mental state. In China’s culture, meeting societal expectations fosters strong self-discipline and attentiveness. Mistakes or lapses in attention, such as those caused by mind wandering, may therefore be perceived not only as personal failures but also as failures to meet the expectations of the group or family. This broader cultural context may help contextualise why the Chinese participants in the current study viewed mind wandering primarily in a negative light, as it conflicts with their cultural values of effort and focus. It seems to be this dimension of cultural difference that matters most in contexts where tasks are implicitly or explicitly tied to self-discipline, or achievement. Indeed, Asian heritage students tend to think that making an effort in learning is a student’s obligation (Chen et al., 2016). Thus, the negative perceptions of mind wandering observed in the Chinese sample is consistent with a cultural framework that prioritise discipline and collective responsibility.

The more varied perceptions of mind wandering among British participants may similarly be interpreted in light of cultural values. An individualistic culture, like the United Kingdom, compared to those from a collectivist culture (e.g., China; See Sun et al., 2004) places a greater emphasis on personal autonomy and independence (Welzel and Inglehart, 2010). Consistent with this orientation, British sample in our study frequently described intentionally permitting mind wandering during unimportant tasks, suggesting a greater sense of control over their own mental processes. This aligns with individualistic cultural values that prioritise their own goals or interests over social expectations.

These evaluative differences likely operate through distinct psychological mechanisms. For Chinese participants, the internalisation of authoritarian educational values may mean mind-wandering triggers guilt for violating a duty, or shame for lacking disciplined self-control. Their self-regulation may be more vigilant against lapses that could be seen as failing contextual expectations. For British participants, a focus on personal agency may foster a more permissive internal dialogue, allowing mind-wandering to be framed as a neutral or strategic mental activity. Thus, while the cognitive occurrence of mind-wandering shows cross-cultural consistency, the cognitive and emotional evaluation of that occurrence is deeply shaped by the cultural constructions of attention, obligation and personal responsibility.

### Limitations

The primary limitations are inherent to the study’s exploratory, qualitative design. The sample size and composition (*n* = 17 UK vs. *n* = 32 China) were determined by the principle of thematic saturation to meet the core aim of developing a taxonomy, not by statistical power for group comparisons. Consequently, this research was not designed for, nor should it be interpreted as, a confirmatory test of cultural hypotheses. This design directly shapes the interpretability of the quantitative results. The numerous null findings in cross-cultural comparisons are uninformative regarding the true absence of an effect, as the study was underpowered to detect subtle differences. The single statistically significant difference in evaluation is best viewed as a preliminary pattern for future verification. Indeed, where comparisons were made, the observed means between groups were consistently very close, suggesting any underlying cross-cultural differences would likely be subtle and associated with a small effect size, requiring a large sample to detect reliably. Therefore, the quantitative analyses are presented for contextual and exploratory purposes only. The study’s primary contribution and value lie in the rich, qualitative insights and the novel taxonomy it generates. These outputs are intended to guide future confirmatory research with larger, balanced samples and direct measures of cultural orientation to quantitatively test the taxonomy’s reliability, and external validity.

A further issue is the use of probes to capture mind wandering. Two methods were employed to log this: self-initiated logging when they noticed their own errors (non-interruptive), and researcher-initiated probes during the task (interruptive). While the use of task-interrupting probes may influence natural task pacing, they were necessary to sample mind wandering that occurs without conscious awareness—episodes that would otherwise be missed in self-caught or retrospective reports. This is especially important given that not all off-task thought leads to observable errors, and some mind wandering occurs beneath the threshold of meta-awareness.

A fundamental constraint of this study lies in the operationalization of culture. While the UK/China comparison served as a valid exploratory test for group-level differences, treating nationality as a proxy for culture is inherently broad. Crucially, this study did not employ individual-level measures of cultural orientation (e.g., individualism–collectivism, analytic-holistic thinking) or specific value systems (e.g., Confucianism). This design limitation has two key interpretive consequences. First, the null findings regarding the frequency and triggers of mind-wandering remain ambiguous; it could indicate genuine cross-cultural similarity or mask opposing within-group heterogeneities. Second, while the observed cross-cultural difference in the evaluation of mind-wandering is coherent with major cultural theories, it cannot causally attribute it to specific cultural dimensions without direct measurement. Therefore, the findings are best interpreted as suggestive patterns. It underscores the necessity for future research to incorporate validated, individual-level cultural instruments to move beyond group-level description and towards individual-level explanatory mechanisms.

## Conclusion

This study revealed mind wandering as a complex phenomenon influenced by both universal cognitive processes and, to a lesser extent, cultural factors. The finding of deliberate mind wandering during an unengaging task challenges the traditional view that it is purely spontaneous. Cultural orientations shaped participants’ evaluation of mind wandering: Chinese participants viewed it more negatively than British participants. However, the experience of mind wandering (frequency, triggers) showed cross-cultural consistency, underscoring its universal cognitive basis.

## Data Availability

The numerical data supporting the findings of this study are available in the article and its Supplementary material. The qualitative interview transcripts are not publicly available due to ethical restrictions and confidentiality agreements with participants. Inquiries regarding data access may be directed to the corresponding author.
